# The Morphology of the Femur Influences the Fracture Risk during Stumbling and Falls on the Hip—A Computational Biomechanical Study

**DOI:** 10.3390/life14070841

**Published:** 2024-07-02

**Authors:** Jan-Oliver Sass, Michael Saemann, Maeruan Kebbach, Ehsan Soodmand, Andreas Wree, Rainer Bader, Daniel Kluess

**Affiliations:** 1 Research Laboratory for Biomechanics and Implant Technology, Department of Orthopaedics, Rostock University Medical Center, Doberaner Str. 142, 18057 Rostock, Germany; 2 Julius Wolff Institut, Center for Musculoskeletal Biomechanics and Regeneration, Berlin Institute of Health—Charité—Universitätsmedizin Berlin, Augustenburger Platz 1, 13353 Berlin, Germany; ehsan.soodmand@gmail.com; 3 Institute for Anatomy, Rostock University Medical Center, Gertrudenstraße 9, 18057 Rostock, Germany

**Keywords:** femur morphology, anatomy, finite-element analysis, bone mechanics, fracture risk

## Abstract

Proximal femur fracture risk depends on subject-specific factors such as bone mineral density and morphological parameters. Here, we aim to analyze the dependency of the femoral strength on sixteen morphological parameters. Therefore, finite-element analyses of 20 human femurs during stumbling and lateral falls on the hip were conducted. Pearson correlation coefficients were calculated and morphological parameters with significant correlations were examined in principal component analysis and linear regression analysis. The dependency of the fracture strength on morphological parameters was more pronounced during lateral falls on the hip compared to stumbling. Significant correlations were observed between the neck shaft angle (r = −0.474), neck diameter (r = 0.507), the true distance between the femoral head center and femoral shaft axis (r = 0.459), and its projected distance on the frontal plane (r = 0.511), greater trochanter height (r = 0.497), and distance between the femoral head center and a plane parallel to the frontal plane containing the projection of the femoral head center to the femoral neck axis (r = 0.669). Principal component analysis was strongly weighted by parameters defining the lever arm during a lateral fall as well as the loaded cross-section in the femoral neck.

## 1. Introduction

Bone diseases of the human musculoskeletal system are a public health challenge [1,2,3]. In particular, fractures of the proximal femur are associated with high mortality and morbidity, and therefore represent a socioeconomic burden [1,3]. A retrospective study reported mortalities after proximal femur fractures of 7% at 30 days, 14.5% at six months, and 21.5% at one year [1]. Furthermore, patients suffer from chronic pain, loss of mobility, and decreased quality of life [1,2,3].

Among younger patients (<50 years), femur fractures mostly occur due to high energy events (e.g., accidents, falls from a great height), whereas in the elderly population low energy events, i.e., stumbling or lateral fall on the hip from a standing position, might lead to a proximal femur fracture [4,5]. In this context, Neto et al. [5] reported that 39% of low-energy femur fractures occurred during moving from sitting to standing up or stair climbing, and approximately 40% occurred while standing still or walking.

The increasing prevalence in the elderly is linked to loss of bone mineral density (BMD) [4,6], which is mainly attributed to osteoporosis. In addition to the overall bone density, the cortical thickness is reduced in osteoporotic femurs [7,8]. Additional factors, such as lack of physical activity, reduced visual contrast sensitivity, a higher probability of falls, and femoral morphology have an influence on the femur fracture risk [6,9,10,11,12,13]. In current clinical practice, the risk of bone fractures is usually assessed according to BMD measurements using dual-energy X-ray absorptiometry (DXA) [9,14]. However, this method shows limited accuracy [15]. Furthermore, fracture risk assessment tools were developed that include several subject-specific characteristics [16].

From a mechanical point of view, the femur morphology determines the moment of inertia and thus contributes to the mechanical response to external loads. In this regard, several previous studies have described the human femur morphology quantitatively [11,17,18,19]. Furthermore, clinical studies have been conducted to identify correlations between femur morphology and the onset of fractures at the proximal femur [11,20,21]. It was shown that the hip axis length, femoral neck angle, and neck width have a significant influence on the fracture risk at the proximal femur [11]. Although some retrospective studies indicated the influence of morphological parameters on the fracture risk [11], they were not able to study influencing parameters comprehensively in standardized and clinically relevant load scenarios, nor were they able to determine high-risk combinations of morphological parameters.

In this context, numerical simulations of the musculoskeletal system such as finite-element (FE) analysis is a feasible computational approach to investigate the mechanical response of bone tissue (stress and strain distributions) to external loads [12,13,22,23,24,25,26], and thus to systematically analyze influencing factors that are contributing to the mechanical strength of the femoral bone [10]. Gong et al. [10] have investigated correlations between morphological parameters and femoral fracture risk deploying FE analysis of a single-leg stance, which is a frequently used load case in the literature [27,28,29,30,31]. As this loading pattern is similar to stumbling and associated with high mechanical stresses, it is relevant for investigating femur fractures [5,10,32]. Viceconti et al. [24] showed that it is crucial to assess the fracture risk in multiple loading scenarios due to highly diverse in vivo fracture loads. Apart from the single-leg stance configuration, lateral falls on the greater trochanter are frequently investigated and are a major cause of fractures in the proximal femur [5,24,33,34,35,36,37,38].

The aim of this computational study was to investigate the influence of several morphological parameters of the human femur on its mechanical strength during stumbling and lateral falls. Therefore, a correlation analysis followed by principal components analysis (PCA) and linear regression analysis was conducted to identify the morphological parameters with the highest influence on femoral strength.

## 2. Materials and Methods

### 2.1. Human Specimens

The study was approved by the ethical committee of the Rostock University Medical Center (application number: A 2019-0164). Accordingly, CT scans of 20 femurs from human specimens (fresh frozen or formalin-fixed) were carried out. Details of the CT datasets of femoral bone specimens are presented in Table 1. The donors were 70.1 ± 16.5 years old (range: 48–92 years), and no information of the sex was given. Based on the CT datasets, 3D models of the human femurs were digitally reconstructed using AMIRA^®^ v.5.4.1 (Thermo Fisher Scientific, Waltham, MA, USA and Zuse Institute Berlin, Berlin, Germany). A semi-automatic segmentation algorithm combining a threshold-based selection of the bone structure and manual editing of the epiphysis regions was employed to reconstruct the 3D surface of the femurs. Hounsfield Units (HU) were used as thresholding criteria, and were individually adapted for each femur to precisely segment the 3D surface. Subsequently, manual selection of cancellous bone and bone marrow was performed. Accordingly, 3D surfaces of the whole bone, trabecular bone, and bone marrow were created.

In a next step, the 3D surfaces were converted into datasets consisting of analytical non-uniform rational B-splines for further morphological characterization and FE analysis in Geomagic Studio v.10 (3D Systems Inc., Rock Hill, SC, USA) [39].

### 2.2. Femoral Morphology Characterisation

**Figure 1:**
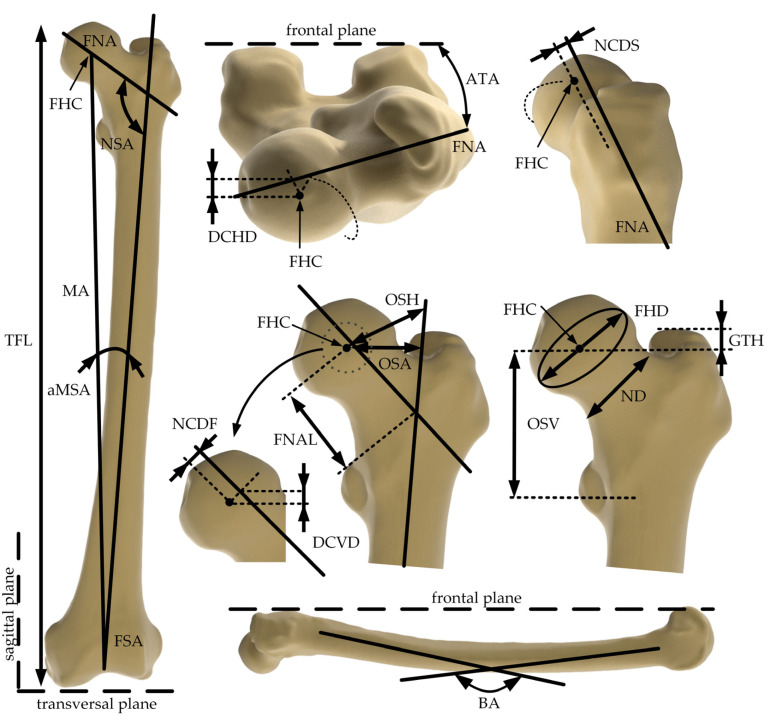
Human femur in different anatomical planes and illustration of anatomical landmarks (femoral shaft axis (FSA), femoral neck axis (FNA), and femoral head center (FHC)) and the well-defined morphological parameters according to Soodmand et al. [17]: femoral antetorsion angle (ATA), angle between FSA and mechanical axis (aMSA), bending angle of the femoral shaft projected on the sagittal plane (BA), distance between the FHC and a plane parallel to the frontal plane containing the projection of the FHC to the FNA, positive for anterior position of the FHC and negative for posterior position (DCHD), vertical distance between the FHC and a plane parallel to transversal plane containing the projection of the FHC to the FNA, positive for cranial positions of the FHC and negative for caudal positions (DCVD), femoral head diameter (FHD), femoral neck axis length (FNAL), vertical distance between FHC and the plane parallel to the transversal plane containing the most proximal point of the greater trochanter (GTH), distance between FHC and FNA projected to the frontal plane (NCDF), distance between FHC and FNA projected to the sagittal plane (NCDS), neck diameter (ND), femoral neck-shaft-angle (NSA), distance between FHC and FSA (OSA), projected distance between FHC and FSA to the frontal plane (OSH), vertical distance between the FHC and the plane parallel to the transversal plane containing the center of the lesser trochanter (OSV), and total femur length (TFL).

Sixteen descriptive morphological parameters were defined, which were previously described [17,18,19]. These morphological parameters are specified in Table 2 and illustrated in Figure 1. The femoral shaft axis (FSA), femoral neck axis (FNA), femoral head center (FHC), as well as frontal, transversal, and sagittal planes were defined as previously described by Soodmand et al. [17] and used as anatomical landmarks. Each parameter was measured six times, and averaged values were used for further analyses.

### 2.3. Finite Element Analysis

#### 2.3.1. General Model Assumptions and Discretization Strategy

**Figure 2:**
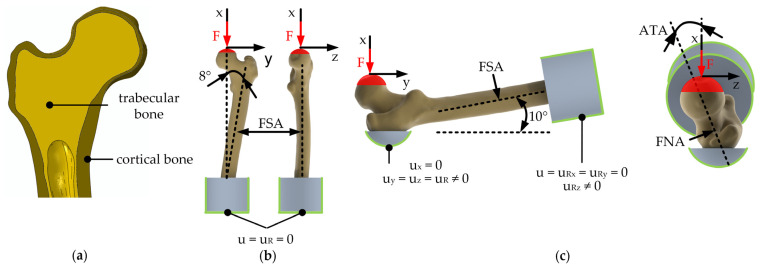
Representative femur with (**a**) reconstructed cortical and trabecular bone geometry. Depiction of the boundary conditions used in terms of force application simulating (**b**) stumbling, and (**c**) a lateral fall. F: applied load; u_x,y,z_: displacements, u_R_: rotation vector, FSA: femoral shaft axis, ATA: antetorsion angle, FNA: femoral neck axis.

Subject-specific, quasi-static FE models of the femurs during stumbling and lateral falls on the hip were established using Abaqus/CAE Standard v6.14 (Dassault Systèmes, Providence, RI, USA) [40] and were generally based on the CT datasets of the femurs. The geometries of the whole femur, trabecular bone, and bone marrow were imported into Abaqus v6.14 for each reconstructed bone. Separate 3D models of the cortical and trabecular bone were created using Boolean operators. The interface between the cortical and trabecular bone was constrained to zero degrees of freedom (tie constraint). The geometrical representation of the cortical and trabecular bone is shown in Figure 2a.

**Figure 3:**
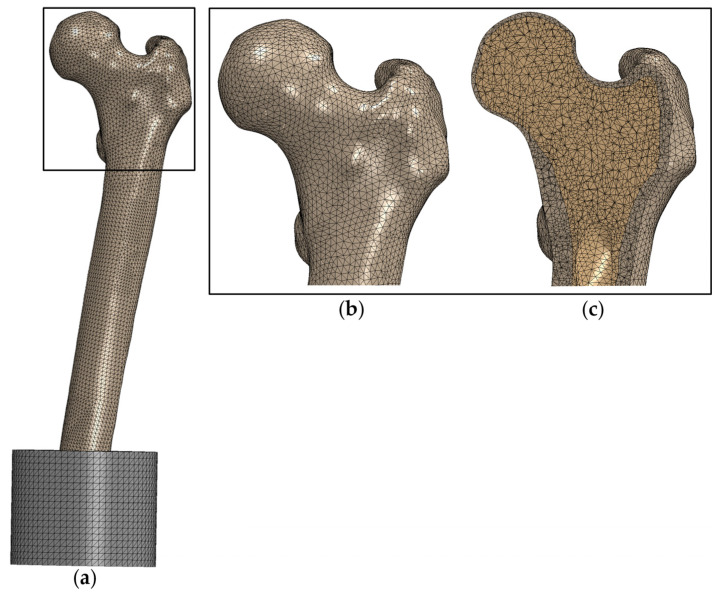
Representative discretized finite element model of (**a**) the whole femur, (**b**) magnified view of the proximal femur and (**c**) section view of the proximal femur.

The model was discretized using quadratic, tetrahedral finite elements (C3D10) with a mean edge length of 2.5 mm (see Figure 3). A mesh convergence analysis for both load cases was conducted for one femur. It was shown that further refinement of the mesh leads to changes in the strength of less than 5%.

#### 2.3.2. Boundary Conditions and Material Properties

The boundary conditions and corresponding degrees of freedom are illustrated in Figure 2b,c. The femurs were cut to two-thirds of the original TFL, and the distal shaft was embedded in a self-curing polymeric cylinder (diameter: 89 mm, height: 70 mm, Young’s modulus: 2400 MPa, Poisson’s ratio: 0.35 (according to manufacturer specifications). The contact surface between the femur and the polymer embedding was constrained to zero degrees of freedom. In both models, the load of 10,000 N was applied in 100 N increments on a reference point, which was kinematically coupled with a hemisphere of the distal FHD 10 mm in height.

Cortical and trabecular bone were each defined as a linear-elastic, isotropic material with a Young’s modulus of 16 GPa and 0.5 GPa, respectively, as well as a Poisson’s ratio of 0.3 [41]. An asymmetric maximum-strain-based failure criterion (ε_tensile_ = 0.0073, ε_compression_ = 0.0104) was adopted to calculate the femoral strength [42]. The first step in which a continuous group of elements in the cortical bone with a total volume of at least 100 mm³ exceeds one of the critical strain limits is defined as a fracture [43].

To simulate stumbling, the femurs were tilted by 8° around the sagittal axis and at 0° around the transversal and vertical axis [29]. A lateral fall on the hip was simulated by aligning the femoral shaft axis by 10° to the vertical axis [37]. As no tilting around the femoral axis took place, the native ATA was used as the angle between the sagittal axis and load axis in the transversal plane. Furthermore, the greater trochanter was embedded in the same self-curing polymer. For this, the displacement was constrained in the vertical direction, which is parallel to the force axis.

#### 2.3.3. Statistical Analysis

Statistical analysis of the calculated mechanical strength and the correlation to the femur morphology was based on Gong et al. [10] and performed using SPSS v25 (IBM Corp., Armonk, NY, USA). Descriptive statistics are presented as mean ± standard deviation. First, pearson correlation coefficients r between the strength and morphological parameters of the femur and between the morphological parameters were calculated and their level of significance determined. Afterwards, a PCA with significant correlation coefficients was conducted. PCA is a statistical technique used to transform high-dimensional data into a lower-dimensional space while preserving as much variance as possible. The identified principal components (PCs) are linear combinations of the original variables. In the context of the data of this present study, the PCA identifies morphological parameters that are majorly contributing to the fracture strength [10]. All requirements for the specific tests were checked within the statistical analysis, and the significance level was set at *p* ≤ 0.05. In the PCA, components with eigenvalues >1 were considered. Finally, a multivariate linear regression analysis was performed to analyze the relationship between PCs and fracture strength.

## 3. Results

**Figure 4:**
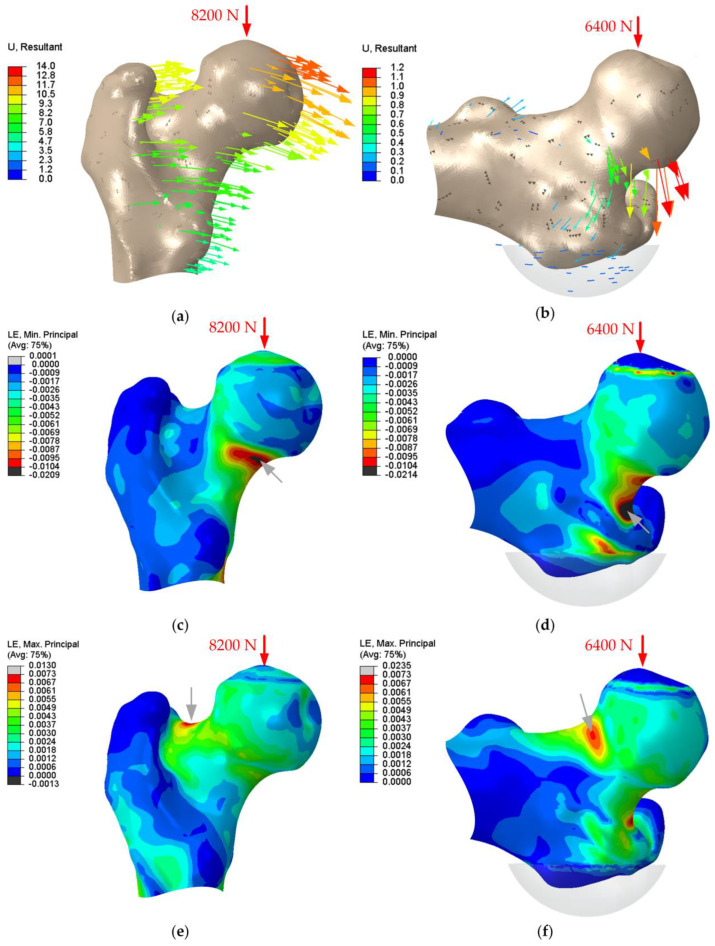
Results of the finite element analysis of one representative human femur (81 years) showing the displacement vectors during (**a**) stumbling and (**b**) a lateral fall, the compressive strain (LE, min principal) during (**c**) stumbling and (**d**) a lateral fall, and the tensile strain (LE, max principal) during (**e**) stumbling and (**f**) a lateral fall. The compressive force vector applied at the femoral head is indicated by a red arrow. The human femur showed a compressive fracture at 8200 N or 6400 N during stumbling or a lateral fall, respectively. The location of potential fracture initiation is marked with a grey arrow.

The FE models were able to reproduce typical displacements and strain distribution for the applied loadings. In Figure 4, the displacement vectors as well as the compressive and tensile strain distribution for one exemplary proximal femur with fracture strengths of 8200 N and 6400 N for stumbling and lateral falls are shown. Black areas indicate the exceeded strain limit and therefore the fracture initiation. For stumbling, the asymmetric strain limits were either reached in the superior or inferior femoral neck for a tensile or compressive fracture, respectively. For lateral falling, all femurs have shown compressive fractures, and the strain limit was exceeded at the superior part of the femoral neck.

In Table 3, the descriptive statistics of the fracture strengths and morphological parameters of the investigated human femurs are summarized.

**Figure 5:**
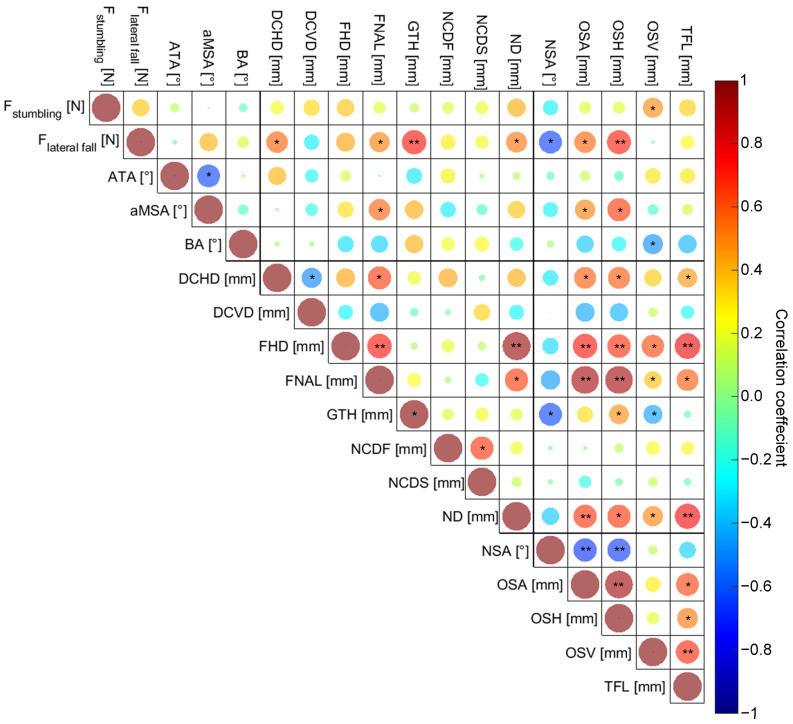
Correlation matrix of the fracture loads during stumbling or lateral fall and the morphological parameters ((femoral antetorsion angle (ATA), angle between FSA and mechanical axis (aMSA), bending angle of the femoral shaft projected on the sagittal plane (BA), distance between the FHC and a plane parallel to the frontal plane containing the projection of the FHC to the FNA, positive for anterior position of the FHC and negative for posterior position (DCHD), vertical distance between the FHC and a plane parallel to transversal plane containing the projection of the FHC to the FNA, positive for cranial positions of the FHC and negative for caudal positions (DCVD), femoral head diameter (FHD), femoral neck axis length (FNAL), vertical distance between FHC and the plane parallel to the transversal plane containing the most proximal point of the greater trochanter (GTH), distance between FHC and FNA projected to the frontal plane (NCDF), distance between FHC and FNA projected to the sagittal plane (NCDS), neck diameter (ND), femoral neck-shaft-angle (NSA), distance between FHC and FSA (OSA), projected distance between FHC and FSA to the frontal plane (OSH), vertical distance between the FHC and the plane parallel to the transversal plane containing the center of the lesser trochanter (OSV), and total femur length (TFL)). Significant correlations are indicated as: * *p* < 0.05, ** *p* < 0.001.

The correlation coefficients and their level of significance are presented in Figure 5, and the exact values are presented in Appendix A. Within the stumbling load case, only the OSV correlated significantly (r = 0.490, *p* = 0.028) with the fracture strength, and therefore PCA was not appropriate. For the lateral fall, DCHD (r = 0.571, *p* = 0.009), FNAL (r = 0.513, *p* = 0.021), GTH (r = 0.728, *p* < 0.001), ND (r = 0.532, *p* = 0.016), NSA (r = −0.641, *p* = 0.002), OSA (r = 0.567, *p* = 0.009), and OSH (r = 0.705, *p* < 0.001) correlated significantly with the fracture strength, and these parameters were therefore used for PCA.

For the dataset of the significantly correlated morphological parameters during a lateral fall, two principle components were extracted (Table 4). The Kaiser-Meyer-Olkin-Criterion (KMO = 0.732) and Bartlett’s test (*p* < 0.001) demonstrated the applicability of the method. The two PCs explained 80.3% of the total variance of the morphological parameters that were significantly correlated with femoral strength.

The weighting of the morphological parameters in the principle components are presented in Table 5. In both PCs, morphological parameters that influence the lever arm between the load axis and the pivot point, i.e., the bearing at the greater trochanter, were identified.

Results of the multivariate linear regression analysis are shown in Table 6. The extracted PCs were significantly predictive for the fracture strength (r = 0.809, *p* < 0.001). The standard error of the prediction was 904.6 N.

## 4. Discussion

Due to the association of proximal femur fractures with high mortality and morbidity [1], it is necessary to identify high-risk patients in a timely manner and initiate therapeutic and preventive measures. Current clinical practice to predict fracture risk represents BMD measurement using dual-energy X-ray absorptiometry [44], whereas several studies have shown correlations between the femur morphology and the subject-specific bone fracture risk [10,11].

Studies in which morphological parameters are separately considered are rare. Therefore, the aim of this study was to determine the impact of femoral morphology on the fracture strength during stumbling and lateral falls. For this purpose, 20 subject-specific FE models of human femoral bones were generated, and sixteen descriptive morphological parameters were measured. Statistical analysis of the association between FE-predicted fracture strength and femur morphology was performed using correlation analysis, PCA, and linear regression analysis.

The boundary conditions of the loading cases were adopted from validated FE models [29,37,45]. The defined strain-based failure criterion [42] was used in several independent studies [10,29]. Comparable studies reported experimental fracture strengths for the load cases for stumbling 6800 ± 904 N [29], 8710 ± 2930 N [45], 6237 ± 1125 N [30], and lateral falls 3120 ± 1140 N [45], 1409 N to 6179 N [34], 3364 ± 1247 N [37]. The results of our present study of 6115 ± 1339 N (3900–8500 N) and 5640 ± 1420 N (2700–8800 N) for stumbling and lateral falls, respectively are in a realistic range; however, this study lacks experimental validation and thus the comparison of the absolute values to other studies is limited. We predominantly observed compressive fractures of the femurs, which was not observed in comparable computational studies [29,35,46]. This might be attributed to the homogenous approach to model the bone’s properties. However, heterogeneous modelling was not appropriate due to changed mineral contents in formalin-fixed human femur specimens. Since this simplification was made for all femurs, we assume that the identification of highly relevant morphological parameters is reliable; however, the fracture pattern observed has to be interpreted with restrictions.

Since the strength during stumbling is higher than during a lateral fall, and only one morphological parameter correlated significantly with the fracture strength during stumbling, it is indicated that the lateral fall loading is more relevant regarding the investigation of femoral fracture risks. This is in line with clinical findings regarding the incidence of femur fracture causes, where 68% are accounted to falls and only 4% to stumbling [5]. However, Gong et al. [10] reported significantly correlated morphological parameters (ND, FHD, OSA) in a stumbling load case, which might be attributed to different FE model assumptions. As the stumbling load case is comparable to a single leg stance [10,29,47], the femur is adapted to this daily load case due to bone remodeling [48]. We therefore assume that unfavorable macroscopic femur morphology, i.e., high FNAL together with low ND, is compensated for by bone remodeling (densification). However, in our FE models, this phenomenon is only accounted for by cortical thickness, not by heterogeneous material distribution.

During a lateral fall, several morphological parameters (DCHD, FNAL, GTH, ND, NSA, OSA, and OSH) correlated significantly with the FE-predicted strength. In line with Soodmand et al. [17], we have also observed inter-correlation between the morphological parameters. Therefore, a PCA was conducted to minimize the influences of parameter interactions and to identify the parameters with the highest contribution to the strength during lateral fall. The first PC was majorly weighted by FNAL, OSA, and OSH, while the second was majorly weighted by GTH. In addition to these parameters that are influencing the lever arm between the load axis and pivot point, an increased ND (weighted with 0.729 in PC-1) leads to a higher cross-section and therefore to an increased load bearing capacity. These findings are consistent with clinical observations, where FNAL and ND have been described to significantly influence the fracture risk at the proximal femur [11]. Accordingly, worst-case combinations of femur morphology are those leading to a high lever arm during a lateral fall; e.g., high FNAL, and a relatively small loaded cross-section in the femoral neck (expressed by ND).

The femur strength is also influenced by the bone quality, i.e., osteoporosis status, which is expressed by the BMD [4,6,10] and cortical thickness [7,8]. These factors were not considered in our present study because the different storage conditions of the femurs (fresh frozen vs. formalin-fixed) made a reliable comparison among the femurs unfeasible. Decreased bone quality is represented within the FE models by the cortical thickness, as the femur geometry of the bone domains (cortical and trabecular bone) were reconstructed from CT images. The advantage of our approach is the isolated investigation of subject-specific morphology without any offsets due to bone densification and quality. Nevertheless, the biological response to mechanical loading over time may compensate for unfavorable morphological parameters or a combination of these [48].

The femur morphology is also sex and age-dependent [49,50], and Jepsen et al. [51] demonstrated that sex differences in femur strength are not only explained by the larger size of the femur in men. They concluded that the differences are based on different structures. Unfortunately, sex and age differences could not be investigated with the current dataset. However, the presented workflow can evaluate this with larger cohorts in the future. Our present study’s results suggest including the morphological parameters DCHD, FNAL, GTH, ND, NSA, OSA, and OSH, and focusing on the lateral fall on the hip.

This study has some limitations. First, the bone material properties were simplified. It is well known that bones have heterogeneous and anisotropic mechanical properties, and FE models can use the relationship between Young’s modulus and mineral density to assign these properties in FEA [15,29,34,41,45]. Since formalin fixation changed the mineral content and thus the HU in CT scans, this method was not applicable within the present dataset. We therefore chose a homogenous approach where the geometry of the cortical and cancellous bone is based on the segmentation of the CT datasets. The applied properties are in the range of previous studies [52,53,54] and have shown good agreement with experimental tests [41]. However, it has to be mentioned that Mohammadi et al. [41] used anisotropic material properties. Stumbling and lateral falls’ dynamic and high-energy events were based on literature studies [29,37] and simplified to a static analysis. However, this approach was also made in a comparable study by Gong et al. [10]. Furthermore, experimental validation of the FE models was not conducted, but we have discussed the FE-predicted fracture strength in regard to comparable studies as recommended by Hicks et al. [55].

The results highlight the importance of including morphological parameters, which significantly contribute to the femoral strength, in future studies of the subject-specific fracture risk of the human femur. Based on the described data, a study containing sufficiently large cohorts for calibration of a regression model could be conducted. In addition to the morphological parameters, proven variables that contribute to the fracture risk should be included like in other fracture risk assessment tools [16].

## 5. Conclusions

Our computational study showed that the fracture strength of the human femoral bone is influenced by morphological parameters with greater effects during lateral falls compared to stumbling. Statistical analysis showed that the principal components are especially weighted by morphological parameters determining the lever arm between the load axis and pivot point (e.g., FNAL, GTH, NSA) or the loaded cross section (ND). In clinical practice, 2D radiographs are more easily available than 3D CT scans. Therefore, the morphological parameters that can be assessed in the frontal plane, i.e., FNAL, GTH, ND, OSH, and NSA might be more relevant for clinicians and present additional factors to consider during fracture risk assessment. With the presented workflow, future studies could focus on gender and age-related differences in femur strength, which should include the significantly correlated parameters determined in this study and focus on the lateral fall on the hip as a representative high-energy load case.

## Figures and Tables

**Table 1:** Description of the CT scans of the human femoral specimens.

Number of Specimens	CT Scanner	Resolution [mm³]	Preparation Process
6	Aquilion 64, Toshiba, Tokyo, Japan	0.4 × 0.4 × 0.5	fresh frozen
4	Brilliance CT Big Bore, Philips AG, Amsterdam, Netherlands	0.4 × 0.4 × 0.5	formalin-fixed
1	SOMATOM Definition AS + CT scanner, Siemens AG, Munich, Germany	0.7 × 0.7 × 1.0	fresh frozen
9		0.3 × 0.3 × 0.6	formalin-fixed

**Table 2:** Abbreviations and descriptions of morphological parameters used in this study according to Soodmand et al. [17] and Bao et al. [18], and the plane or method used to determine the parameters where the anatomical landmarks femoral head center (FHC), femoral shaft axis (FSA), and femoral neck axis (FNA) were used.

Abbreviation [unit]	Explanation	Determination Method
ATA [°]	femoral antetorsion angle	transversal plane
aMSA [°]	the angle between the mechanical axis and FSA projected on the frontal plane	frontal plane
BA [°]	the bending angle of the femoral shaft projected on the sagittal plane	sagittal plane
DCHD [mm]	distance between the FHC and a plane parallel to the frontal plane containing the projection of the FHC to the FNA, positive for the anterior position of the FHC and negative for the posterior position	transversal plane
DCVD [mm]	the vertical distance between the FHC and a plane parallel to the transversal plane containing the projection of the FHC to the FNA; positive for cranial positions of the FHC and negative for caudal positions	frontal plane
FHD [mm]	femoral head diameter	best fit sphere
FNAL [mm]	distance from the intersection of the FSA and FNA to the FHC (representing the ideal lever arm)	frontal plane
GTH [mm]	the vertical distance between FHC and the plane parallel to the transversal plane containing the most proximal point of the greater trochanter	frontal plane
NCDF [mm]	distance between FHC and FNA projected to the frontal plane	frontal plane
NCDS [mm]	distance between FHC and FNA projected to the sagittal plane	sagittal plane
ND [mm]	neck diameter projected on the frontal plane	frontal plane
NSA [°]	femoral neck-shaft-angle	frontal plane
OSA [mm]	distance between FHC and FSA	3-dimensional
OSH [mm]	projected distance between FHC and FSA in the frontal plane	frontal plane
OSV [mm]	the vertical distance between the FHC and the plane parallel to the transversal plane containing the center of the lesser trochanter	frontal plane
TFL [mm]	total femoral length	frontal plane

**Table 3:** Mean values, standard deviation (SD), minimum, and maximum values of the fracture strength for stumbling (F_stumbling_) and lateral falls (F_lateral fall_) and the morphological parameters (femoral antetorsion angle (ATA), angle between FSA and mechanical axis (aMSA), bending angle of the femoral shaft projected on the sagittal plane (BA), distance between the FHC and a plane parallel to the frontal plane containing the projection of the FHC to the FNA, positive for anterior position of the FHC and negative for posterior position (DCHD), vertical distance between the FHC and a plane parallel to transversal plane containing the projection of the FHC to the FNA, positive for cranial positions of the FHC and negative for caudal positions (DCVD), femoral head diameter (FHD), femoral neck axis length (FNAL), vertical distance between FHC and the plane parallel to the transversal plane containing the most proximal point of the greater trochanter (GTH), distance between FHC and FNA projected to the frontal plane (NCDF), distance between FHC and FNA projected to the sagittal plane (NCDS), neck diameter (ND), femoral neck-shaft-angle (NSA), distance between FHC and FSA (OSA), projected distance between FHC and FSA to the frontal plane (OSH), vertical distance between the FHC and the plane parallel to the transversal plane containing the center of the lesser trochanter (OSV), and total femur length (TFL)) of the human femurs (the donors were 70.1 ± 16.5 years old (range: 48–92 years)).

Parameter		Mean	SD	Minimum	Maximum
Fracture strength					
F_stumbling_	[N]	6115	1339	3900	8500
F_lateral fall_	[N]	5640	1420	2700	8800
Morphological parameters					
ATA	[°]	10.26	5.64	1.99	24.44
aMSA	[°]	5.24	1.31	3.27	8.78
BA	[°]	11.19	2.62	4.44	15.75
DCHD	[mm]	1.13	1.98	−3.75	5.11
DCVD	[mm]	−0.19	1.26	−1.68	2.25
FHD	[mm]	48.74	4.04	42.48	55.76
FNAL	[mm]	51.90	7.23	36.05	64.15
GTH	[mm]	7.89	5.01	−0.27	17.57
NCDF	[mm]	2.00	0.93	0.54	4.40
NCDS	[mm]	2.12	0.89	0.67	4.37
ND	[mm]	38.02	3.35	32.11	44.83
NSA	[°]	124.84	4.56	115.15	134.62
OSA	[mm]	45.71	6.41	30.49	59.12
OSH	[mm]	41.85	7.09	27.47	58.53
OSV	[mm]	59.31	5.68	49.41	71.37
TFL	[mm]	468.44	37.71	396.42	531.31

**Table 4:** Extracted principle components (PCs) of the significant correlated morphological parameters during lateral fall.

PC	Eigenvalue	Percentage of Variance	Cumulative Percentage of Variance
1	4.5	63.7	63.7
2	1.2	16.7	80.4

**Table 5:** Weighting of the morphological parameters (distance between the femoral head center and a plane parallel to the frontal plane containing the projection of the femoral head center to the femoral neck axis, positive for anterior position of the femoral head center and negative for posterior position (DCHD), femoral neck axis length (FNAL), vertical distance between femoral head center and the plane parallel to the transversal plane containing the most proximal point of the greater trochanter (GTH), neck diameter (ND), femoral neck-shaft-angle (NSA), distance between FHC and FSA (OSA), projected distance between femoral head center and femoral shaft axis to the frontal plane (OSH) in the extracted principle components.

Parameter	PC-1	PC-2
DCHD	0.680	0.298
FNAL	0.915	0.293
GTH	0.499	−0.776
ND	0.729	0.277
NSA	−0.724	0.547
OSA	0.954	0.111
OSH	0.971	0.004

**Table 6:** Multivariate regression model of the extracted principal components (PC-1, PC-2) and the fracture strength during lateral fall.

Model	Unstandardized Coefficient		Standardized Coefficient	*p*-Value
	Regression Coefficient B	Standard Error	Beta	
Constant	5640.0	202.3		0.000
PC-1	710.1	207.5	0.5	0.003
PC-2	940.8	207.5	0.6	0.000

## Data Availability

Data will be made available upon reasonable request.

## Appendix A

**Table A1:** Pearson correlation coefficients between morphological parameters (femoral antetorsion angle (ATA), angle between FSA and mechanical axis (aMSA), bending angle of the femoral shaft projected on the sagittal plane (BA), distance between the FHC and a plane parallel to the frontal plane containing the projection of the FHC to the FNA, positive for anterior position of the FHC and negative for posterior position (DCHD), vertical distance between the FHC and a plane parallel to transversal plane containing the projection of the FHC to the FNA, positive for cranial positions of the FHC and negative for caudal positions (DCVD), femoral head diameter (FHD), femoral neck axis length (FNAL), vertical distance between FHC and the plane parallel to the transversal plane containing the most proximal point of the greater trochanter (GTH), distance between FHC and FNA projected to the frontal plane (NCDF), distance between FHC and FNA projected to the sagittal plane (NCDS), neck diameter (ND), femoral neck-shaft-angle (NSA), distance between FHC and FSA (OSA), projected distance between FHC and FSA to the frontal plane (OSH), vertical distance between the FHC and the plane parallel to the transversal plane containing the center of the lesser trochanter (OSV), and total femur length (TFL)) and fracture strengths during stumbling (r_stumbling_) and a lateral fall (r_lateral fall_) as well as their level of significance (*p*), with significant correlations (*p* ≤ 0.05).

Parameter		r_stumbling_	p_stumbling_	r_lateral fall_	p_lateral fall_
ATA	[°]	0.099	0.677	−0.027	0.911
aMSA	[°]	−0.003	0.992	0.401	0.080
BA	[°]	−0.090	0.706	0.163	0.492
DCHD	[mm]	0.212	0.369	0.571	0.009
DCVD	[mm]	0.324	0.164	−0.294	0.208
FHD	[mm]	0.370	0.108	0.430	0.059
FNAL	[mm]	0.181	0.445	0.513	0.021
GTH	[mm]	0.114	0.632	0.728	<0.001
NCDF	[mm]	0.163	0.493	0.283	0.227
NCDS	[mm]	0.203	0.390	0.212	0.369
ND	[mm]	0.418	0.067	0.532	0.016
NSA	[°]	−0.285	0.223	−0.641	0.002
OSA	[mm]	0.172	0.469	0.567	0.009
OSH	[mm]	0.187	0.430	0.705	0.001
OSV	[mm]	0.490	0.028	−0.016	0.946
TFL	[mm]	0.349	0.131	0.230	0.328
